# Isolation and Genetic Characterization of Mangshi Virus: A Newly Discovered *Seadornavirus* of the *Reoviridae* Family Found in Yunnan Province, China

**DOI:** 10.1371/journal.pone.0143601

**Published:** 2015-12-02

**Authors:** Jinglin Wang, Huachun Li, Yuwen He, Yang Zhou, Jingxing Meng, Wuyang Zhu, Hongyu Chen, Defang Liao, Yunping Man

**Affiliations:** 1 Yunnan Tropical and Subtropical Animal Viral Disease Laboratory, Yunnan Animal Science and Veterinary Institute, Kunming, Yunnan province, China; 2 State Key Laboratory of Veterinary Etiological Biology, Lanzhou, Gansu province, China; 3 State Key Laboratory for Infectious Disease Prevention and Control, National Institute for Viral Disease Control and Prevention, Chinese Center for Disease Control and Prevention, Beijing, China; The Pirbright Institute, UNITED KINGDOM

## Abstract

**Background:**

*Seadornavirus* is a genus of viruses in the family *Reoviridae*, which consists of *Banna virus*, *Kadipiro virus*, and *Liao ning virus*. *Banna virus* is considered a potential pathogen for zoonotic diseases. Here, we describe a newly discovered *Seadornavirus* isolated from mosquitos (*Culex tritaeniorhynchus*) in Yunnan Province, China, which is related to *Banna virus*, and referred to as *Mangshi virus*.

**Methods and Results:**

The *Mangshi virus* was isolated by cell culture in *Aedes albopictus* C6/36 cells, in which it replicated and caused cytopathic effects, but not in mammalian BHK-21 or Vero cells. Polyacrylamide gel analysis revealed a genome consisting of 12 segments of double-stranded RNA, with a “6–4–2” pattern in which the migrating bands were different from those of the *Banna virus*. Complete genome sequencing was performed by full-length amplification of cDNAs. Sequence analysis showed that seven highly conserved nucleotides and three highly conserved nucleotides were present at the ends of the 5′- and 3′-UTRs in each of 12 genome segments. The amino acid identities of *Mangshi virus* shared with *Balaton virus* varied from 27.3% (VP11) to 72.3% (VP1) with *Banna virus* varying from 18.0% (VP11) to 63.9% (VP1). Phylogenetic analysis based on amino acid sequences demonstrated that *Mangshi virus* is a member of the genus *Seadornavirus* and is most closely related to, but distinct from, *Balaton virus* and *Banna virus* in the genus *Seadornavirus* of the family *Reoviridae*.

**Conclusion:**

*Mangshi virus* isolated from mosquitoes (*C*. *tritaeniorhynchus*) was identified as a newly discovered virus in the genus *Seadornavirus* and is phylogenetically close to *Banna virus*, suggesting that there is genetic diversity of seadornaviruses in tropical and subtropical areas of Southeast Asia.

## Introduction

There are currently three identified species of the genus *Seadornavirus* within the family *Reoviridae* including *Banna virus* (BAV), *Kadipiro virus* (KDV), and *Liao ning virus* (LNV), whose genomes consist of 12 segments of double-stranded RNA (dsRNA) [1,2]. BAV is the prototype species of the genus *Seadornavirus* and was first isolated in 1987 from patients with encephalitis in southern China, Yunnan Province, Xishuangbanna Prefecture [2,3]. Since then, BAV was also isolated from mosquitoes, pigs, cattle, and ticks in China [4,5], Indonesia [6], and Vietnam [7], indicating that BAV is pathogenic to humans and may be an emerging pathogen or perhaps responsible as an undiagnosed cause of flu-like symptoms and viral encephalitis in humans in some areas of these countries [8].

*Balaton virus*, representing a novel *Seadornavirus* species, was identified in the intestinal contents of freshwater carp (*Cyprinus carpio*) in Hungary using viral metagenomics [9]. However, the virus was not isolated from carp and only eleven complete genome segments encoding VP1, VP2, VP3, VP4, VP6, VP7, VP8, VP9, VP10, VP11, and VP12 of the virus were determined. In this study, a virus strain (DH13M041), referred to as *Mangshi virus*, was isolated from mosquitoes (*C*. *tritaeniorhynchus*) collected in the southwest of Yunnan Province, China. The virus was found to have a genome composed of 12-segmented dsRNA. Here, we describe the molecular properties of the virus and its phylogenetic relationship with members of *Seadornavirus*. Our data indicate that *Mangshi virus* is a new *Seadornavirus* species akin to *Balaton virus* and BAV.

## Materials and Methods

### Ethics statement

Authorization for the collection of mosquitoes was obtained from the Institute for Yunnan Animal Science and Veterinary Institute, Kunming, China (protocol approval number: 201303035). The institution approved oral consent for collection before initiating the project entitled “Isolation and Genetic Characterization of *Mangshi Virus*: A Newly Discovered *Seadornavirus* of the *Reoviridae* Family Found in Yunnan Province, China” considering that the target population lives in rural areas and they receive little education. The purpose of the survey was explained to district administrative authorities, and then to village leaders and a veterinarian in the location before sample collection, and their agreement were obtained. Before mosquito collections, the investigators explained the objectives of mosquito collection to the head of each household and they provided their informed oral consent with the assistance of the village leaders and the rural veterinarian. No clinical investigation was conducted and no personal identifiers were included; therefore, no written informed consent was sought for this study.

### Cell cultures

BHK-21 and Vero cells were grown in minimal essential medium (MEM; HyClone, USA) with a balanced salt solution supplemented with 10% fetal bovine serum (FBS), 100 U/ml of penicillin, and 100 μg/ml of streptomycin. The mammalian cells were propagated and maintained at 37°C under an atmosphere of 5% CO_2_. C6/36 (*Aedes albopictus*) cells were propagated and maintained in 60% Dulbecco’s modified Eagle’s medium (HyClone) plus 30% Roswell Park Memorial Institute 1640 (HyClone) with Hanks’ salt solution supplemented with 10% FBS, 100 U/ml of penicillin, and 100 μg/ml of streptomycin, at 28°C under an atmosphere of 5% CO_2_.

### Mosquito collection and virus isolation

Mosquito samples were collected from caprine and bovine shelters at night using light traps (12 V, 300 mA; Wuhan Lucky Star Environmental Protection Tech Co., Hubei, China) in a suburb of Menga Town (24°19′27″N, 98°32′13″E, altitude = 1156 m) in Mangshi City, Dehong Prefecture, southwestern Yunnan Province during July 2013. Light traps were set on overnight from 7:00 pm to 7:00 am until the next morning, i.e., from sunset to sunrise. Captured mosquitoes were killed by freezing at –20°C for at least 30 minutes and were collected on a chilled plate. Mosquito species were identified by using morphologic characteristics. Each ≤100 mosquitoes of the same species were pooled in a cryogenic vial and stored in liquid nitrogen until virus isolation was conducted.

Pools of mosquitoes were homogenized in 1 ml grinding fluid (MEM with 100 U/ml of penicillin and 100 μg/ml of streptomycin, pH 7.4) in a sterile glass grinder and centrifuged in an Eppendorf tube at 5000*g* for 10 min, and then 200 μl supernatant was inoculated into each well containing a monolayer of C6/36, Vero, or BHK-21 cells in 24-well plates respectively for 7 days. Further blind passages were repeated three times. The cells were observed daily for cytopathic effects (CPE) and the cell culture supernatants were collected and stored at –80°C for further analysis.

### Polyacrylamide gel electrophoresis (PAGE) and RT-PCR

Viral RNA was extracted from infectious C6/36 cells using RNAiso Plus (TaKaRa, Dalian, China) according to the manufacturer’s instructions. The RNA was subjected to electrophoresis at room temperature on a standard discontinuous 7%, 10%, or 15% acrylamide (acrylamide/bisacrylamide 29:1; Bio-Rad Laboratories, Hercules, CA) slab gel (18 cm × 16 cm × 0.075 cm) (Hoefer Pharmacia Biotech Inc., San Francisco, CA) with a 3.5% acrylamide stacking gel in Tris–glycine buffer (25 mM Tris, 192 mM glycine, pH 8.3) (Bio-Rad Laboratories). After electrophoresis, virus dsRNA was visualized by staining with silver nitrate as described previously [10,11].

First-strand cDNA was synthesized using PrimeScript^®^ Reverse Transcriptase (TaKaRa) according to the manufacturer’s instructions. The viral cDNA was subjected to polymerase chain reaction (PCR) amplification with primers targeting the 12^th^ segment of BAV [12]. Amplified products were separated by electrophoresis on 1% agarose gel in TAE buffer (40 mM Tris–acetate, 1 mM Ethylenediaminetetraacetic acid (EDTA), pH 8.0) with nucleic acid dye (GoldView), and the result was observed under ultraviolet light.

### Full genome sequencing

Genome sequencing was performed by full-length amplification of cDNAs (FLAC) as described previously [13]. Briefly, the isolates were propagated in C6/36 cells in 75 cm^2^ tissue culture flasks. Total RNA was extracted from the infected cells with greater than 90% CPE using RNAisoPlus (TaKaRa) according to the manufacturer’s instructions. Single-stranded RNA (ssRNA) was removed by precipitation with 2 M LiCl (Sigma) at 4°C for 16 h and the dsRNA was purified by addition of 2.5 volumes of isopropanol and 1 volume of 7.5 M ammonium acetate, followed by two more washes with 70% ethanol, and resuspended in RNase-free water. The dsRNA was subjected to 1% agarose gel electrophoresis (AGE) (7 V/cm, for 1 h) in TAE buffer and purified from the agarose gel using a MinElute gel extraction kit (Qiagen).

An “anchor primer” PC3-T7 loop (synthesized by Sangon Biotech, Shanghai, China), similar to that described by Maana et al. [14], was used in ligation. The “anchor primer” PC3-T7 loop (100 ng) was ligated to viral dsRNA (100 ng) and ligation reactions were conducted in a total volume of 30 μl as described previously [13, 14]. The ligated dsRNA was purified using MicroElute RNA Clean Up Kit (Omega) based on the manufacturer’s directions. The virus dsRNA was denatured by the addition of dimethyl sulfoxide to a final concentration of 15% (v/v), heated in boiling water for 2 min, and snap-frozen in ice-water slurry. The full-length cDNA of 12 viral dsRNA segments was synthesized using a High Fidelity PrimeScript II RT-PCR kit (TaKaRa) according to the manufacturer’s instructions. The cDNA was annealed at 65°C for 1 h. Amplification of cDNA was conducted with primer PC2 in a total volume of 50 μl as described previously [13,14]. The PCR products were viewed after separation on 1% agarose gels in TAE buffer containing nucleic acid dye, and purified by using a DNA Fragment Purification Kit (version 2.0; TaKaRa). The gel-purified fragments were cloned into the pMD19-T Vector (TakaRa), and then transformed into chemically competent *Escherichia coli* DH5α cells (TakaRa). The selected transformed colonies were cultured and sequenced by the Huada Genome Institute (Shanghai, China).

### Sequence analysis and phylogenetic comparisons

Initial sequence assembly and analysis were conducted by using the ATGC software package (version 4.0; Genetyx, Tokyo, Japan). Sequences were identified by BLAST analysis (http://www.ncbi.nlm.nih.gov/BLAST/). Identity and alignment analysis were analyzed by using the CLUSTAL X (version 2) and MegAlign (DNASTAR, Madison, WI, USA). MEGA 5.1 was used for phylogenetic analysis and tree construction based on the neighbor-joining assay. The bootstrap value (the number of replications) was 1,000. The potential glycosylation sites of the amino acid sequence were determined by an online analysis using NetNGlyc 1.0 (http://www.cbs.dtu.dk/services/).

## Results

### Virus isolation

**Fig 1:**
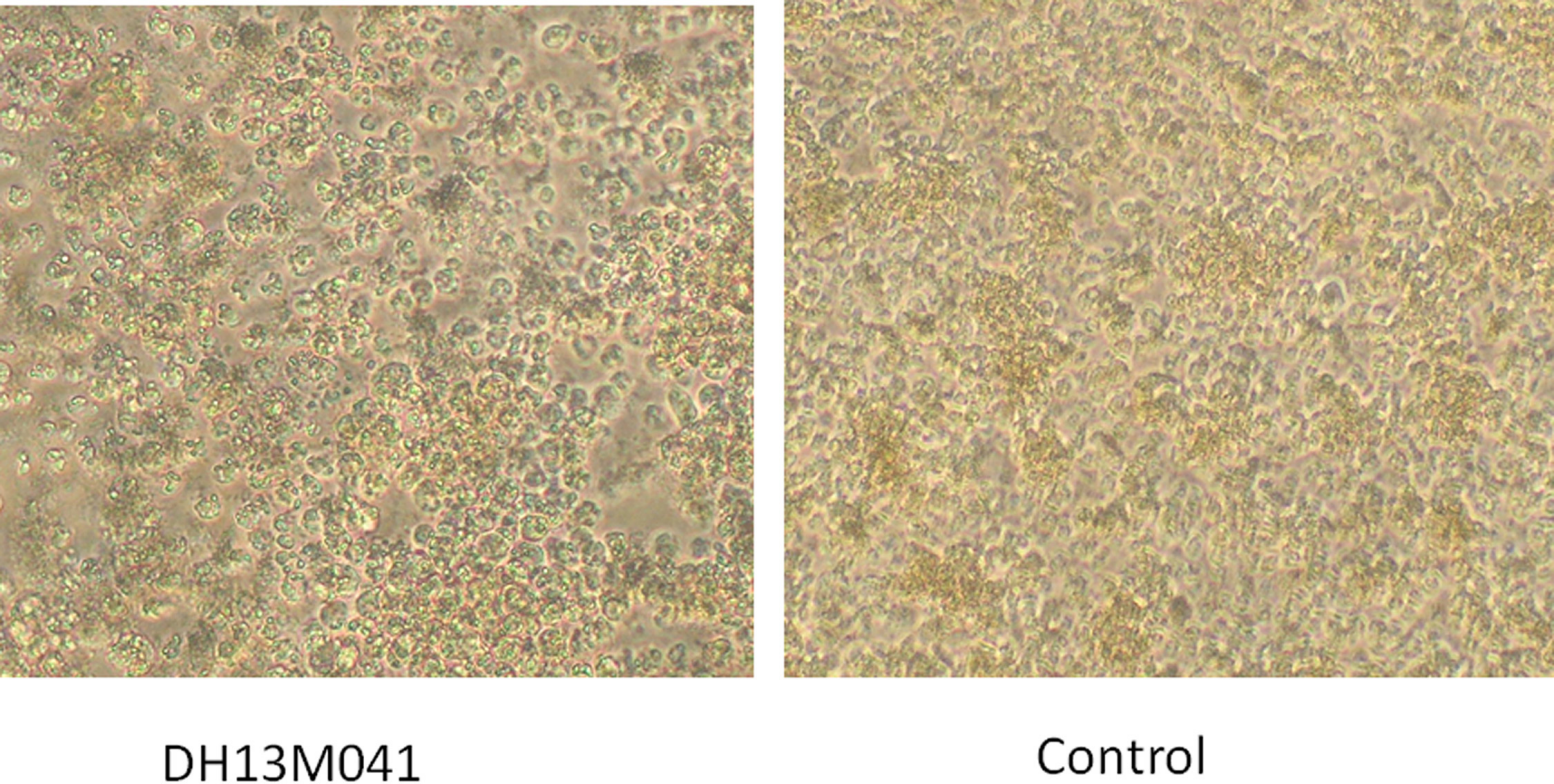
CPE caused by *Mangshi virus* (DH13M041) on C6/36 cells 72 h postinoculation (100×).

The *Mangshi virus* DH13M041 strain was isolated from one pool of mosquitoes (*C*. *tritaeniorhynchus*) captured in Dehong Prefecture, Yunnan Province, China in 2013. The virus was found to cause CPE in second-blind passage in C6/36 cells at 72 h after inoculation. The characteristics of the CPE include cell shrinking, shedding, or cytolysis with eventual detachment from the growth surface (Fig 1). No CPE was observed in mammalian BHK-21 or Vero cells even after three rounds of blind passage at 14 days interval per passage.

### Virus identification by PAGE and RT-PCR

**Fig 2:**
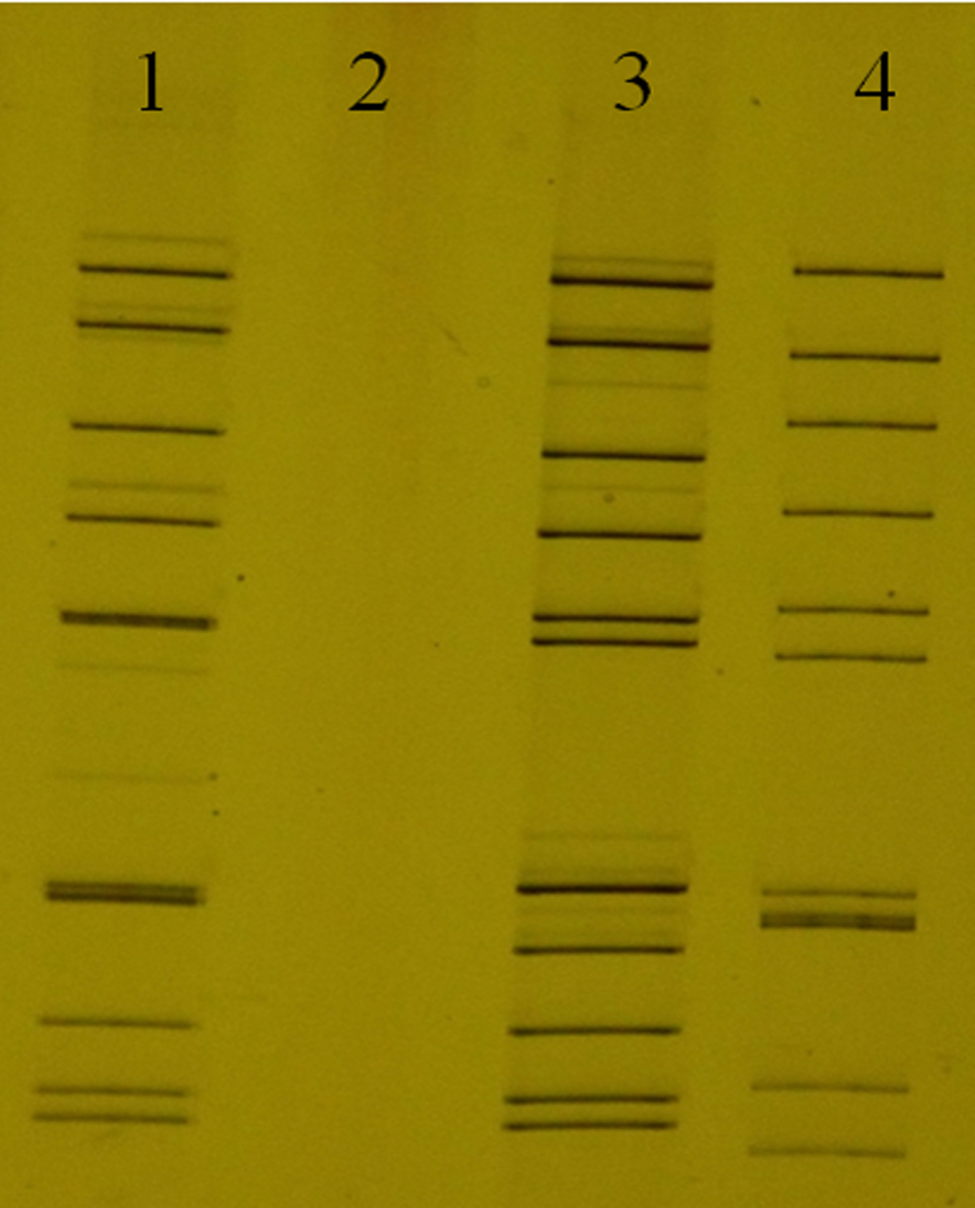
Electrophoretic migration patterns of the dsRNA of *Mangshi virus* (DH13M041) as determined by polyacrylamide gel electrophoresis. 1: DH13M127-6 (BAV); 2:C6/36 Cells control; 3: DH13C233-5 (BAV); 4:DH13M041 (*Mangshi virus*).

**Fig 3:**
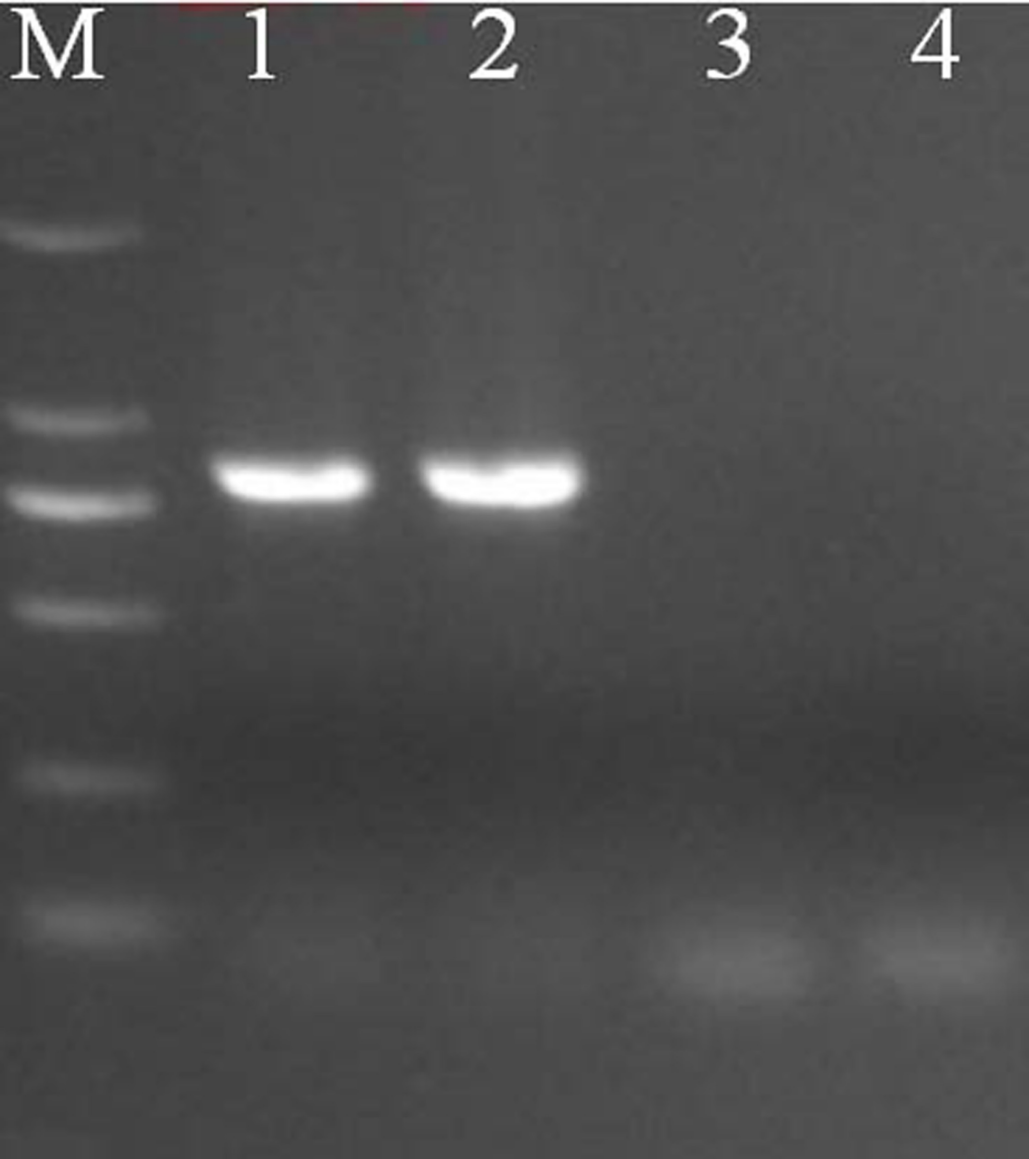
PCR Identification of DH13M041 virus in the culture supernatant of C6/36 cells using primers derived from segment 12 of BAV by 1% agarose gel electrophoresis (AGE). 1: DH13M127-6; 2: DH13C233-5; 3: DH13M041. 4: Negative control.

The dsRNA of Mangshi virus, purified from infected cell cultures, was analyzed by PAGE, along with the RNAs of two BAV previously isolated in Yunnan Province in 2013 and stored in our laboratory, including genotypes A1 BAV DH13M127-6 and A2 BAV DH13C233-5 (Fig 2). PAGE results demonstrated that the *Mangshi virus* is a 12-segmented dsRNA virus with migrating bands different to those of genotypes A1 and A2 of BAV. The majority of segments of the *Mangshi virus* genome migrate separately, with the exception of Seg-8, Seg-9, and Seg-10, which comigrate in this gel system, forming a “6–4–2” pattern. By contrast, both genotype A1 and A2 of BAV show “6–6” patterns. Compared with A1 and A2 BAV, the majority of migrating band segment locations of *Mangshi virus* showed a certain degree of similarity, but is distinguished from the A1 and A2 BAV. In addition, PCR failed to amplify the cDNA from *Mangshi virus* using primers derived from segment 12 of BAV, which successfully amplified cDNA from two BAV strains (A1 genotype BAV DH13M127-6 and A2 genotype BAV DH13C233-5) (Fig 3). These findings suggest that *Mangshi virus* DH13M041 strain is distinct from BAV.

### Genome organization and characteristics of *Mangshi virus*

Like other members of the *Seadornavirus* genus, the *Mangshi virus* has a genome consisting of 12 dsRNA segments. The length of 1–12 segments varies from 788bp (Seg12) to 3740bp (Seg1) and their corresponding encoding proteins and obtained GenBank accession numbers were presented in Table 1. Sequence analysis of the 5′ and 3′ noncoding regions (NCRs) showed that the *Mangshi virus* shares a stretch of seven highly conserved nucleotides and three highly conserved nucleotides at the ends of the 5′- and 3′-UTRs (5′-GUAAAA and GAC-3′, respectively) in each of the 12 gene segments. The first two nucleotides of the 5′-UTRs end are the reverse compliments to the last two nucleotides of the 3′-UTRs end. Each gene segment contains a unique open reading frame and encodes its corresponding protein (VP1–VP12).

**Table 1:** Lengths of dsRNA segments 1 to 12, encoded putative proteins, 5′ and 3′ NCRs of the *Mangshi virus* (DH13M041) genome.

Segment	Length(bp)	Protein(aa)	5′ NCR(bp)	Terminal sequence(5′–3′)	3′ NCR(bp)	GenBank Accession no.
Seg-1	3740	1215	23	GUAAAGAAU------------ACCUGAC	72	KR349187
Seg-2	3055	956	88	GUAAAGAAU------------AAACGAC	99	KR349188
Seg-3	2410	725	24	GUAAAGAAU------------AACUGAC	211	KR349189
Seg-4	2055	628	26	GUAAAGAAU------------GAACGAC	145	KR349190
Seg-5	1718	448	124	GUAAAGAUU------------GACUGAC	250	KR349191
Seg-6	1618	468	31	GUAAAGAAU------------AACUGAC	183	KR349192
Seg-7	1122	298	31	GUAAAGAUU------------GACUGAC	197	KR349193
Seg-8	1114	314	39	GUAAAGAAU------------AACUGAC	133	KR349194
Seg-9	1076	283	23	GUAAAGAUU------------GACCGAC	201	KR349195
Seg-10	1052	254	27	GUAAAGAAA------------GACCGAC	260	KR349196
Seg-11	874	203	41	GUAAAGAAU------------AACUGAC	221	KR349197
Seg-12	788	199	74	GUAAAGAAU------------AAAUGAC	114	KR349198
		Consensus		GUAAAGAWW————-RMMYGAC		

### The identity of amino acid between the Mangshi virus and other seadornaviruses

The identity of amino acid sequence between the Mangshi virus and *Balaton virus* isolated from Europe is from 27.3% (VP11) to 72.3% (VP1) and the identity of amino acid sequence between the *Mangshi virus* and BAV varies from 18.0% (VP11) to 63.9% (VP1). Much lower levels of amino acid identity of the *Mangshi virus* were shared with the KDV and LNV of *Seadornavirus* genus, which vary from 17.3% (LNV VP9) to 40.6% (LNV VP1) (Table 2). The identity of amino acids between nonstructural protein VP8 (protein kinase) encoded by Seg 8 of the *Mangshi virus* and corresponding protein of *Balaton virus* is 49.5%, and corresponding protein of BAV encoded by Seg 7 is from 39.9 to 43.6%. The outer membrane protein VP9 encoded by Seg 9 of the *Mangshi virus* shared the identity of 43% with corresponding protein of *Balaton virus*, and from 34.8 to 36.5% with that of BAV.

**Table 2:** The identity of amino acid between the Mangshi virus (DH13M041) and other *Seadornaviruses*.

Segment*	Balaton	BAV	KDV	LNV	Putative function/location of DH13M41 proteins
Seg-1 (VP1)	Seg-1 (VP1) 72.3%	Seg-1 (VP1) 62.3–63.9%	Seg-1 (VP1) 40.4%	Seg-1 (VP1) 40.1–40.6%	RNA-dependent RNA polymerase/subcore
Seg-2 (VP2)	Seg-2 (VP2) 66.9%	Seg-2 (VP2) 53.6–54.2%	Seg-2 (VP2) 34.0%	Seg-2 (VP2) 35.6–35.9%	T2 layer of core/subcore
Seg-3 (VP3)	Seg-3 (VP3) 56.0%	Seg-3 (VP3) 49.9–50.7%	Seg-3 (VP3) 34.8%	Seg-3 (VP3) 35.9–36.3%	Guanylyltransferase/subcore
Seg-4 (VP4)	Seg-4 (VP4) 61.3%	Seg-4 (VP4) 47.3–48.3%	Seg-4 (VP4) 32.8%	Seg-4 (VP4) 9.8–30.1%	Outer coat protein
Seg-5 (VP5)	Seg-6 (VP6) 44.8%	Seg-6 (VP6) 41.0–41.9%	Seg-5 (VP5) 30.3%	Seg-5 (VP5) 21.4–22.1%	Nonstructural
Seg-6 (VP6)	Seg-7 (VP7) 54.1%	Seg-5 (VP5) 43.9–44.5%	Seg-6 (VP6) 25.4%	Seg-6 (VP6) 25.2%	Nonstructural
Seg-7 (VP7)	Seg-9 (VP9) 60.7%	Seg-8 (VP8) 39.7–41.1%	Seg-9 (VP9) 27.8%	Seg-8 (VP8) 22.3–23.0%	T13 protein/outer layer of core
Seg-8 (VP8)	Seg-8 (VP8) 49.5%	Seg-7 (VP7) 39.9–43.6%	Seg-7 (VP7) 27.6%	Seg-7 (VP7) 21.8–23.8%	Nonstructural (Protein kinase)
Seg-9 (VP9)	Seg-9 (VP9) 43.0%	Seg-9 (VP9) 34.8–36.5%	Seg-11 (VP11) 23.2%	Seg-10 (VP10) 17.3–18.6%	Cell attachment, internalization/outer coat
Seg-10 (VP10)	NA	Seg-10 (VP10) 36.6–38.7%	Seg-10 (VP10) 26.9%	Seg-9 (VP9) 24.1–24.6%	Core protein
Seg-11 (VP11)	Seg-11 (VP11) 27.3%	Seg-12 (VP12) 18.0–20.2%	Seg-8 (VP8) 18.5%	Seg-11 (VP11) 18.1–18.7%	dsRNA binding/non-structural
Seg-12 (VP12)	Seg-12 (VP12) 47.0%	Seg-11 (VP11) 36.3–39.8%	Seg-12 (VP12) 20.6%	Seg-12 (VP12) 18.2–19.6%	Nonstructural

Note

*VP1–VP12 corresponding to Seg-1 to Seg-12 of the Mangshi virus (DH13M041).

The proteins encoded by the *Mangshi virus* 1–12 genome segments were matched with proteins encoded by seadornaviruses by analyzing the deduced amino acid sequence. However, the putative function of each gene segment of the *Mangshi virus* is not consistent with that of the BAV, in which only six proteins encoded by Seg-1, S eg-2, Seg-3, Seg-4, Seg-9, and Seg-10 of the *Mangshi virus* are similar to that of the BAV, and another six proteins encoded by Seg5, Seg6, Seg7, Seg8, Seg11, and Seg12 of the *Mangshi virus* matched the proteins encoded by Seg-6, Seg-5, Seg-8, Seg-7, Seg-12, and Seg-11 of BAV respectively (Table 2).

### Identification of putative viral enzymes and potential glycosylation sites

The sequence analysis of the deduced amino acid showed that there are two conserved motifs SGELTT (positions 710–715) and GDD (positions 755–757) in VP1 of *Mangshi virus*. As is the case for the Balaton virus, BAV, LNV, and KDV [15], VP1 is most likely the viral RdRp and should be designated as VP1 (Pol).

**Fig 4:**
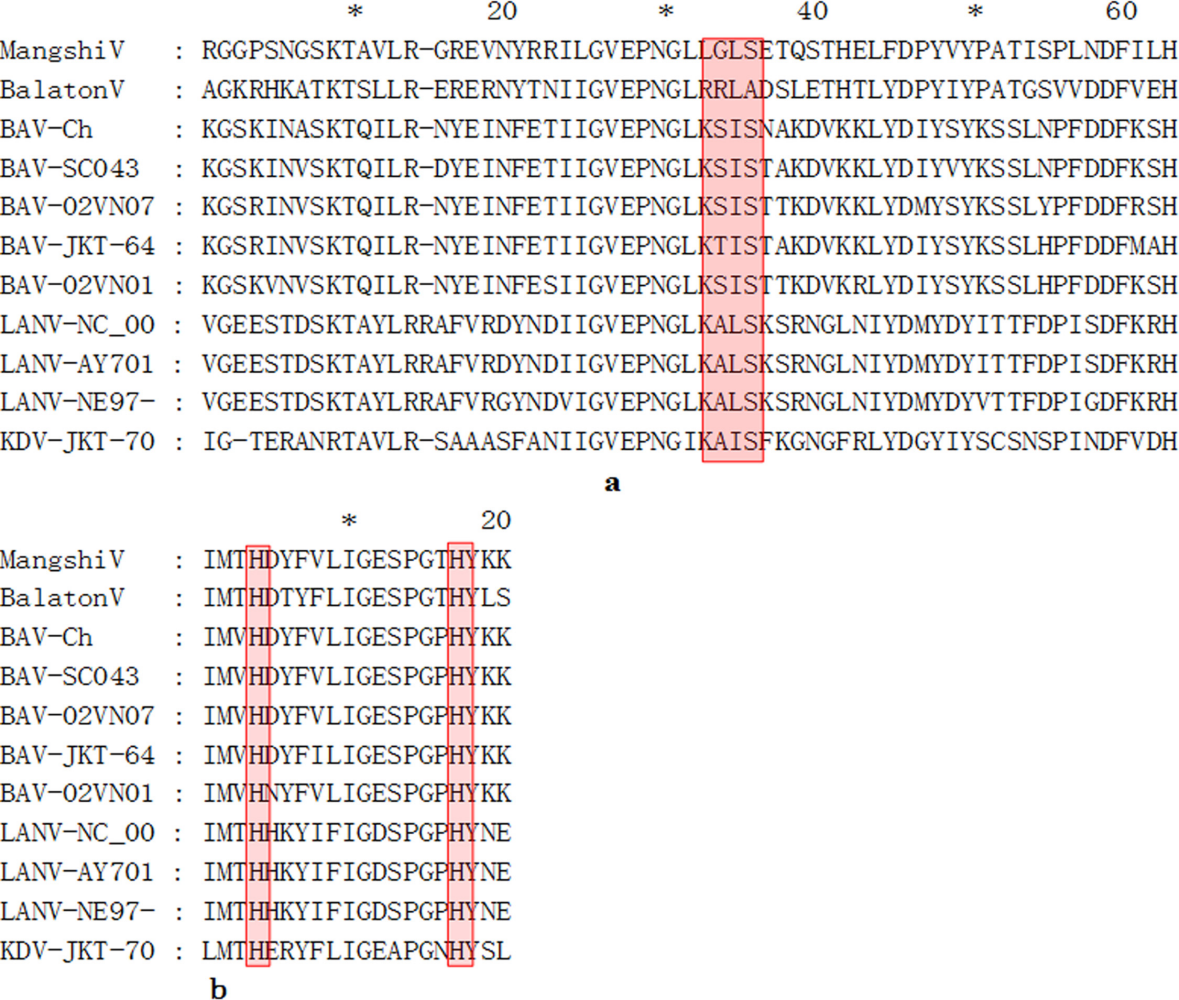
Alignment of the sequence of guanylyltransferases of members of the family *Reoviridae* in the *Mangshi virus* (DH13M041) VP3 with other *Seadornaviruses*. (a) Alignments of sequences of guanylyltransferases in the vicinity of the motif Kx(I/V/L)S is shown in bold characters. Similar sequences are shaded. (b) Alignments of sequences of guanylyltransferases in the vicinity of the two histidine residues (bold) involved in the guanylyltransferase activity in BAV, KDV, and LNV. Similar sequences are shaded.

The identity of the amino acid sequence of VP3 between the *Mangshi virus* and BAV varies from 49.9 to 50.7%. Compared with VP3 of the *Mangshi virus* and VP3 of other seadornaviruses, the Motif Kx(I/V/L)S related to guanylyltransferases is mutated, in which the first aa K is changed into L (Fig 4). Recent study based on orthoreoviruses and aquareoviruses discovered that two histidine residues in VP3 are strongly related to guanylyltransferase activity [16]. These two histidine residues are identical among the *Mangshi virus*, *Balaton virus*, BAV, KDV, and LNV, indicating that they are conserved in the *Seadornavirus* genus.

The host range and virulence of virus as well as host immune response against virus are affected by glycosylation of virus proteins [17]. Analysis of the potential glycosylation sites of the amino acid sequence of viral structural proteins (outer coat protein) showed that there are three glycosylation sites in VP4 and only one in VP9 of the *Mangshi virus*, while there are five glycosylation sites in VP4 and only one in VP9 of BAV (BAV-CH). The identity of amino acids in VP4 between the *Mangshi virus* and BAV is 47.3%–48.3%, and is 34.8%–36.5% in VP9, which suggests that glycosylation sites and numbers of *Mangshi virus* are distinct from BAV.

### Phylogenetic classification of the *Mangshi virus*

**Fig 5:**
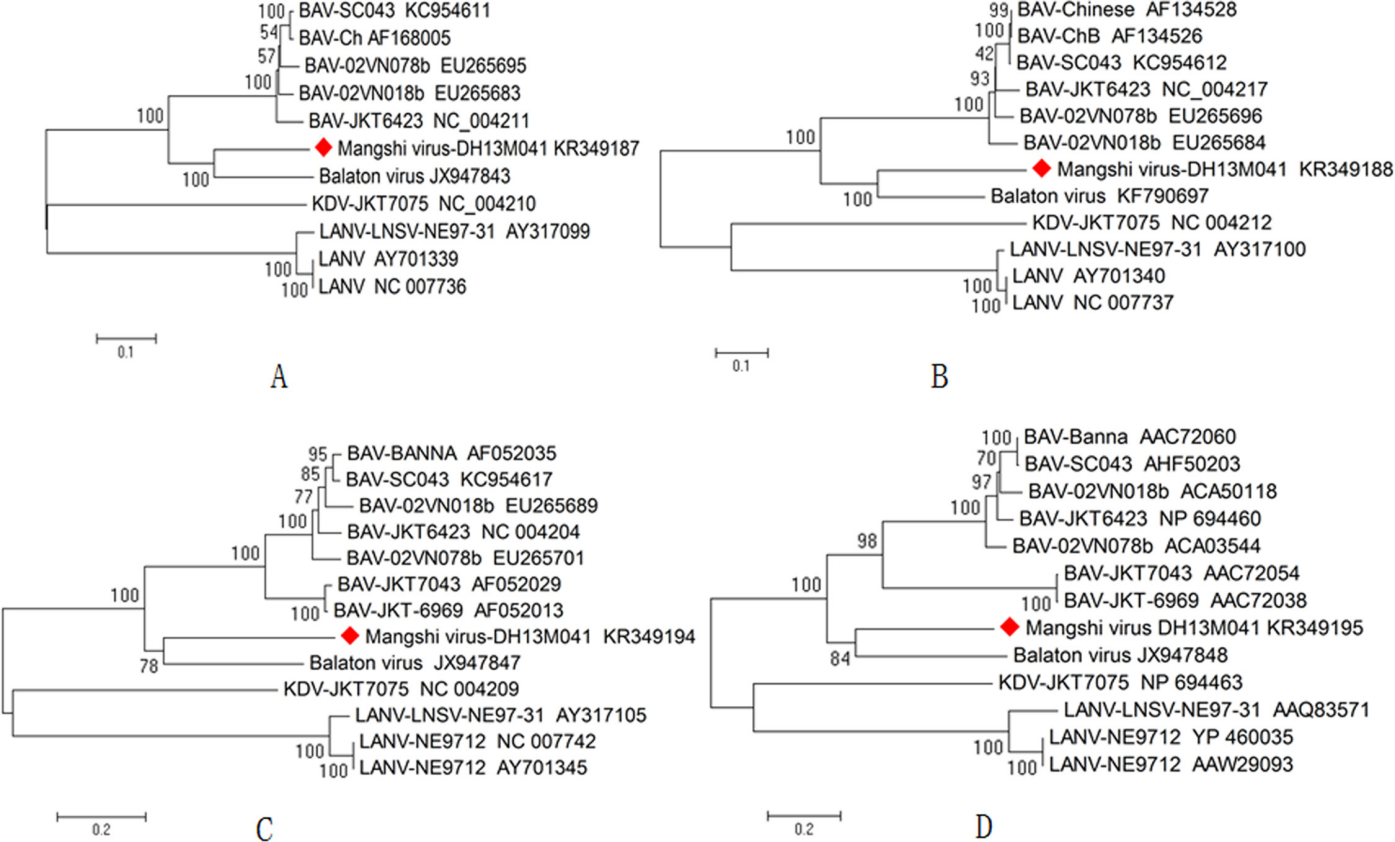
Phylogenetic analysis of complete amino acid sequences of corresponding viral proteins (VP, see Table 1) of *Mangshi virus* (strain DH13M041) using representative members of the species *Balaton virus*, *Banna virus* (BAV), *Kadipiro virus* (KDV) and *Liao ning virus* (LNV) in the genus *Seadornavirus*. (A) RNA-dependent RNA polymerase (VP1); (B) T2 layer of core/subcore (VP2); (C) protein kinase (VP8); (D) outer-coat attachment protein (VP9) of *Mangshi virus*.

Phylogenetic trees based on the amino acid sequences of VP1, VP2, VP8, and VP9 of the *Mangshi virus* and the corresponding sequences from seadornaviruses (*Balaton virus*, BAV, KDV, and LNV) were generated. The results indicate that *Mangshi virus* belongs to the genus *Seadornavirus*. A more detailed analysis indicates that the *Mangshi virus* is most closely related to, but distinct from, Balaton virus and BAV within cluster (Fig 5), suggesting that the *Mangshi virus* is a newly discovered member of the genus *Seadornavirus*.

## Discussion

According to the 8^th^ and 9^th^ meeting report of the International Committee on the Taxonomy of Viruses (ICTV), a new 12-segmented RNA virus genus, *Seadornavirus* genus, was added into the *Reoviridae* family [1,2]. The new genus includes BAV, KDV and LNV, among which, BAV and LNV were both first discovered in China [1,2]. In this study, the *Mangshi virus* isolated from *C*. *tritaeniorhynchus* in Dehong Prefecture, Yunnan Province, is similar to BAV, an important pathogen in viral encephalitis [8], such as only producing CPE in C6/36 cells, trinucleotide motif in the ends of the 3′-UTRs (GAC) and amino acid motifs SGELTT and GDD in VP1. However, many features of the *Mangshi virus* are distinct from those of BAV, including time to develop CPE in C6/36 cells (72 h, which is faster than BAV), migration patterns of viral genome, motif Lx(I/V/L)S in VP3, motif in the ends of the 5′-UTRs as well as glycosylation sites in VP4 and VP9. The gene in segment 11 of the *Mangshi virus* could not be amplified by the specific primers based on the conserved sequence of BAV segment 12 because of the low sequence identity. These data together suggest that the *Mangshi virus* is significantly different from BAV.

Viral RdRp polymerase protein (VP1) encoded by segment 1 is a highly conserved and an important marker for species identification within the family *Reoviridae* (VP1 identity is more than 30% at amino acid level) [18,19]. Within a given *Seadornavirus* species, the identity of VP1 at the amino acid level is from 93% to 99% in BAV [15]. In this study, the identity of VP1 amino acid between the *Mangshi virus* and seadornaviruses including *Balaton virus*, BAV, LNV, and KDV is from 40.1% to 72.3% (more than 30%, but less than 93%), suggesting that the *Mangshi virus* is a newly discovered species of *Seadornavirus*. In addition, the amino acid identity observed in T2 protein (used to classify serotypes within the *Orbivirus* genus) encoded by segment 2 of *Mangshi virus* shares only 34%–66.9% with that of *Balaton virus*, BAV, LNV, and KDV, which is well below the 91% threshold for virus serotype classification according to previously defined criteria of the *Orbivirus* genus [18,20,21]. The phylogenetic trees based on the amino acid sequences of VP1, VP2, VP4, and VP9 demonstrate that the *Mangshi virus* clusters into an independent evolutionary branch in seadornaviruses, indicating that the *Mangshi virus* is a new member of the *Seadornavirus* genus.

BAV is divided into genotype A (BAV-Ch and BAVIn6423) and genotype B (BAVIn6969 and BAV-In7043) by whole-genome sequence analysis. The identity of amino acid sequences of Seg-7 and Seg-9 between two genotypes are 72% and 54%, respectively, and the identity of amino acid sequences among the rest of segments are from 83% to 98% [1]. The amino acid sequence identities of *Mangshi virus* VP8 and VP9 to homologous proteins of *Balaton virus* from Europe are only 49.5% and 43%, and are only 39.9%–43.6% and 34.8%–36.5% to that of BAV, in which all are far less than 72% and 54% (differences between two genotypes of BAV), respectively. The amino acid identity of the other segments (27.3%–72.3% and 18.0%–63.9% in *Balaton virus* and BAV, respectively) is also much less than the threshold (83%) for classification of two genotypes [1]. These findings suggest that the *Mangshi virus* is a newly discovered virus species that is related to *Balaton virus* and BAV.

BAV was firstly isolated from cerebrospinal fluid and sera from patients with viral encephalitis in Banna prefecture of Yunnan Province in 1987. Subsequently, BAV, LNV, KDV, and *Balaton virus* were isolated in various areas of China, Vietnam, Indonesia, and Hungary [7,8,10]. In 2005, a 12-segmented dsRNA virus (MX6) was isolated from *Culex* spp. found at the borders of China, Laos, and Myanmar [11]. Electrophoretic migration patterns of the *Mangshi virus* are similar to that of the MX6 virus, but the identity of amino acid sequences of Seg9 between the MX6 and *Mangshi virus* is only 81%, suggesting that the *Mangshi virus* and MX6 virus may be the same virus of different genotypes.

In summary, we isolated a 12-segmented dsRNA virus from *C*. *tritaeniorhynchus* and demonstrated that it is a new virus member of *Seadornavirus* genus, in the *Reoviridae* family, related to *Balaton virus* and BAV. This is the first report of isolation of a new *Seadornavirus* from *C*. *tritaeniorhynchus* in the southwest border area of Yunnan Province, China. The present study suggests that a number of *Seadornavirus* species and genotypes are prevalent. This should therefore prompt further investigations for a better understanding of the origin and replication of the *Mangshi virus* and the association between the *Mangshi virus* and human public and domesticated animal health.
